# BRAF^V600E^ maintains the CpG island methylator phenotype, and DNA methylation of PRC2 targets genes in colon cancer

**DOI:** 10.1016/j.isci.2025.112905

**Published:** 2025-06-14

**Authors:** Layla El Bouazzaoui, Jeroen M. Bugter, Emre Küçükköse, André Verheem, Jasmin B. Post, Nicola Fenderico, Inne H.M. Borel Rinkes, Hugo J.G. Snippert, Madelon M. Maurice, Onno Kranenburg

**Affiliations:** 1 Division of Imaging and Cancer, Laboratory Translational Oncology, UMC Utrecht, Utrecht, the Netherlands; 2 Oncode Institute and Center for Molecular Medicine, UMC Utrecht, Utrecht, the Netherlands; 3 Utrecht Platform for Organoid Technology, Utrecht University, Utrecht, the Netherlands

**Keywords:** Molecular Genetics, Epigenetics, Cancer, Transcriptomics

## Abstract

In colon cancer, the BRAF^V600E^ mutation is strongly associated with the CpG island methylator phenotype (CIMP). Here, we characterized the contribution of BRAF^V600E^ to maintenance of aberrant DNA methylation using CRISPR-LbCpf1-corrected *BRAF* (V600E) organoids. DNA methylation analyses identified 5,187 differentially methylated CpGs within CpG islands—82% hypermethylated in BRAF^V600E^ organoids—including CIMP-associated genes and polycomb repressor complex 2 (PRC2) target genes. RNA sequencing showed concordant repression of these genes. Furthermore, BRAF^V600E^ organoids demonstrated high expression of PRC2 core components (EZH2, SUZ12, and EED), showed PRC2-induced H3K27 trimethylation in promoter regions, and maintained a PRC2-associated embryonic phenotype. This phenotype was lost following mutation correction or DNA methylation inhibition. These findings show that BRAF^V600E^ maintains aberrant DNA and histone methylation patterns in advanced colon cancer, likely preserving the transformed phenotype. Silencing of PRC2 target genes may contribute to this phenomenon. Epigenetic therapies may have value in the treatment of BRAF^V600E^-mutant colon cancer.

## Highlights

• Correction of BRAF^V600E^ leads to demethylation and de-repression of CIMP-H genes
• BRAF^V600E^ maintains hypermethylation of PRC2 target genes in CRC
• Epigenetic therapies may counteract the aggressive phenotype of BRAF^V600E^ mutant CRC

## Graphical abstract

**Figure:**
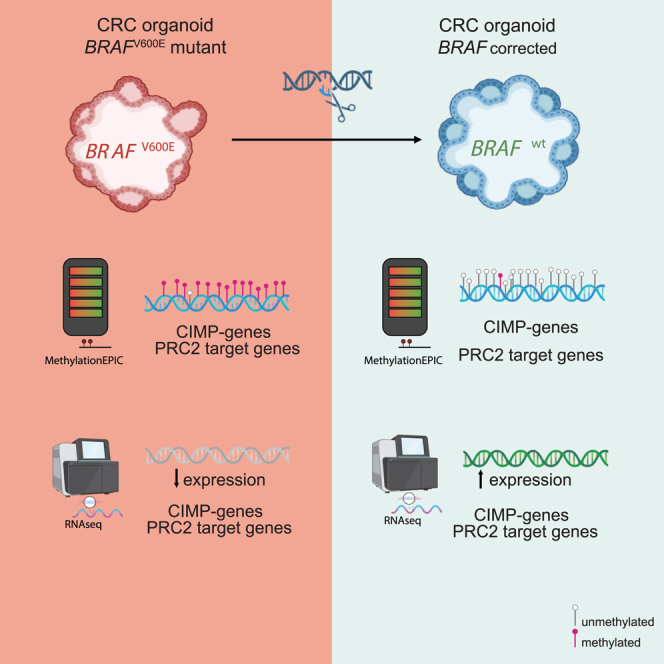

## Introduction

The epigenetic landscape in cancer is generally characterized by DNA hypermethylation of CpG-rich regions known as CpG islands (CGIs).^1^ Hypermethylated CGIs within promoter regions of tumor suppressor genes lead to transcriptional silencing necessary for malignant transformation.^2^ Widespread hypermethylation of a distinct subset of CpG sites, known as the CpG island methylator phenotype (CIMP), is associated with malignant progression in various tumor types and well characterized in colorectal cancer (CRC). CIMP target genes typically overlap with polycomb repressor complex (PRC2) target genes.^3^^,^^4^ PRC2 induces transcriptional silencing by catalyzing the trimethylation of lysine27 (K27) on histone H3 (H3K27me3) in the nucleosomes surrounding promoters.^5^ Embryonic stem (ES) cells rely on PRC2 to repress genes required for differentiation that are poised to be activated during development.^6^ Embryonic PRC2 target genes are more prone to undergo *de novo* DNA methylation than non-targets in human cancers.^7^^,^^8^^,^^9^ PRC2 gene hypermethylation contributes to an aggressive embryonic stem (ES) cell-like phenotype associated with poor prognosis.^10^^,^^11^

CIMP is tightly associated with the BRAF^V600E^ mutation in CRC.^12^^,^^13^ The BRAF^V600E^ mutation induces CIMP through phosphorylation of the transcriptional repressor MAFG, leading to the recruitment of a repressor complex that mediates genome-wide promoter methylation.^14^ In addition, recent mutant BRAF-V600E knock-in models showed that prolonged high BRAF signaling induces a CIMP-like phenotype.^15^ These studies demonstrate a direct causal role for the BRAF^V600E^ mutation in the acquisition of epigenetic changes in CRC. However, other reports show that spontaneous (as opposed to BRAF^V600E^-induced) DNA methylation that occurs as a result of aging,^16^ facilitates subsequent transformation by mutant BRAF^V600E^.^17^^,^^18^ Indeed, the presence of activating mutations in BRAF and CIMP are both correlated with an older age in CRC patients, suggesting that CIMP-like processes occur prior to the acquisition of an oncogenic mutation in BRAF, and facilitate subsequent epithelial transformation.^19^ Additionally, induction of activating mutations in BRAF in cell or organoid models makes them prone to senescence and differentiation,^17^^,^^20^ suggesting that the BRAF^V600E^ mutation is not the first event leading to oncogenesis. Taken together, the relationship between BRAF^V600E^ and CIMP in CRC is complex, and the mechanisms underlying this association are not fully understood.

The question remains whether oncogenic DNA methylation changes are a series of random events resulting in permissiveness to transformation by a BRAF^V600E^ mutant clone,^17^ or whether oncogenic DNA methylation changes are the result of an instructive program induced by the BRAF^V600E^ mutation itself.^14^ In the latter scenario, DNA methylation changes would depend on the continued presence of the BRAF^V600E^ mutation once an aggressive tumor has been established. In the former scenario, this would not be the case. To address this issue, we investigated how established oncogenic epigenetic alterations, such as aberrant DNA methylation, depend on the continued presence of the V600E mutation in BRAF. To this end, we corrected this mutation in colon cancer organoids derived from a late-stage tumor, using the CRISPR-LbCpf1 system. Analysis of the subsequent changes in genome-wide DNA methylation and gene expression revealed that the V600E mutation in BRAF plays a significant role in sustaining the characteristic epigenetic aberrations associated with CIMP and for maintaining an aggressive ES cell-like transformed phenotype. Finally, pharmacological inhibition of DNA methylation strongly suppressed the aggressive phenotype of BRAF^V600E^ colon cancer organoids and resulted in durable treatment responses in BRAF^V600E^ CRC organoids.

## Results

### Correction of the BRAF^V600E^ mutation causes genome-wide DNA demethylation

To explore the BRAF^V600E^-dependency of genome-wide DNA methylation, a BRAF^V600E^ patient-derived organoid (PDO) (HUB040) and two independently generated HUB040 derivatives were employed in which CRISPR technology was used to correct the V600E mutation in BRAF back to E600V.^21^ These PDOs are designated BRAF-corrected 1 (BC1) and BC2. In addition, a non-corrected (NC) PDO was used that underwent the same selection procedure as BC1 and BC2, but in which mutation correction had failed. The same models were used in the accompanying paper where we showed a regained dependency on epidermal growth factor (EGF) receptor signaling, loss of tumorigenic potential, and a role for the BRAF^V600E^ mutation in WNT pathway activation.^21^

**Figure 1:**
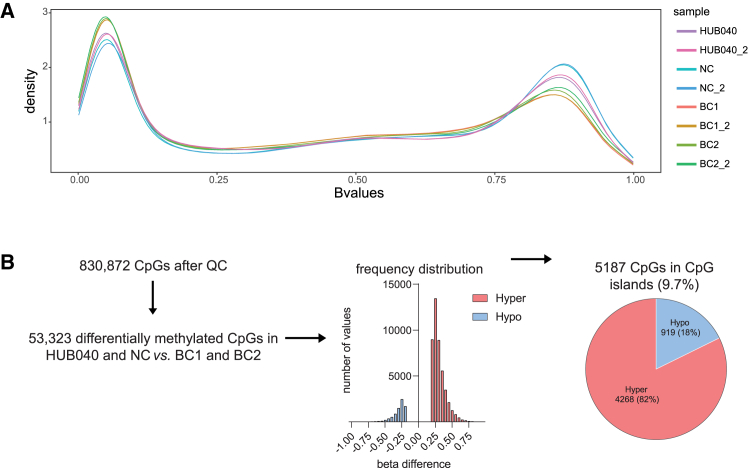
Correction of the BRAF-V600E mutation causes genome-wide DNA demethylation (A) Density plot showing the distribution of B-values (methylation value: 0 = not methylated, 1 = fully methylated) measured at each DNA methylation probe in BRAF^V600E^ mutant (HUB040 and NC) and BRAF^E600V^ (BC1 and BC2) organoids. Two technical replicates per PDO were generated. (B) Distribution of the identified differentially methylated CpGs between HUB040 and NC vs. BC1 and BC2, 85% hypermethylated (45,504 CpGs) and 15% hypomethylated (7819 CpGs) in HUB040 and NC (FDR <0.05, |Δβ| >0.2). 9.7% of these CpGs were located in CpG islands, which showed a similar distribution of hyper- and hypomethylation in HUB040 and NC vs. BC1 and BC2. See also Figure S1 and Table S1. FDR, false discovery rate; QC, quality control.

Genome-wide DNA methylation analysis revealed that hypermethylated regions occurred at higher frequency in BRAF^V600E^ organoids (HUB040 and NC), while hypomethylated regions occurred more frequently in BRAF^E600V^ organoids (BC1 and BC2) (Figures 1A and S1).

DNA methylation analysis led to the identification of 53,323 (of 830,872 CpGs tested, 6.4%) differentially methylated CpGs (false discovery rate [FDR] <0.05, |Δβ| >0.2). Of these, 85% (45,504 CpGs) showed hypermethylation in HUB040 and NC (vs. BC1 and BC2), while 15% (7819 CpGs) showed hypomethylation in HUB040 and NC (vs. BC1 and BC2) (Figure 1B and Table S1). We next focused on CpGs located in CGIs, as methylation of CGIs is principally associated with long-term transcriptional gene repression.^22^ Within CGIs 5187 differentially methylated CpGs were identified between HUB040 and NC versus BC1 and BC2 (Figure 1B and Table S1). Of these, 4268 CpGs (82%; representing 1751 genes) were hypermethylated, and 919 CpGs (18%; representing 450 genes) were hypomethylated in HUB040 and NC (vs. BC1 and BC2).

### Increased expression of DNA methylation and chromatin remodeling factors in BRAF-V600E mutant organoids

Next, RNA sequencing (RNA-seq) analysis was performed on HUB040, NC, BC1, and BC2 organoids. We assessed the expression of key epigenetic modifiers (Figure S2). RNA-seq analysis revealed significant upregulation of UHRF1, HDAC2, MBD3, MBD4, SUZ12, EZH2, and EED in BRAF-V600E mutant organoids (HUB040 and NC) compared to BRAF-corrected organoids (BC1 and BC2). UHRF1, crucial for maintaining DNA methylation, recruits DNMT1 to replication sites, suggesting its upregulation enhances DNMT1 activity and contributes to global hypermethylation. Elevated levels of SUZ12, EZH2, and EED, components of the PRC2 complex, imply increased H3K27me3 deposition, promoting a repressive chromatin environment. HDAC2, MBD3, and MBD4, part of chromatin remodeling complexes, facilitate histone deacetylation and link DNA methylation to histone modifications, further contributing to gene silencing and chromatin remodeling in BRAF*-*V600E mutant organoids. Interestingly, the NC organoid line displayed higher hypermethylation levels than HUB040, which may be attributed to elevated DNMT expression, potentially reflecting a subclonal adaptation with a higher baseline level of DNA methylation. To validate these findings more broadly, we analyzed the expression of these key epigenetic modifiers in larger colorectal cancer datasets, including the Marisa et al.^23^ dataset and the TCGA COAD^24^ cohort (Figure S3). Consistent with our observations, EZH2, EED, and UHRF1 were significantly upregulated in BRAF-mutant CRCs compared to BRAF-wildtype CRCs in these datasets.

### BRAF^V600E^ maintains the CIMP-H phenotype

**Figure 2:**
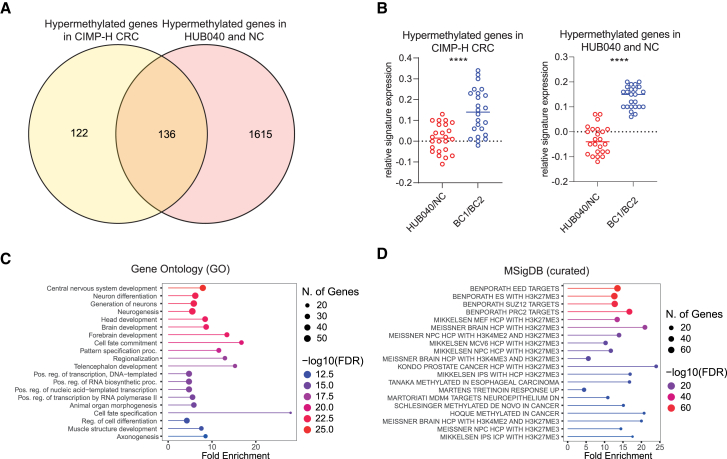
BRAF^V600E^ maintains the CIMP-H phenotype (A) Overlap of CIMP-H geneset (*n* = 258) and genes annotated to hypermethylated CpGs within CGIs (*n* = 1,751) obtained from comparison of HUB040 and NC vs. BC1 and BC2. (B) Relative signature expression of hypermethylated genes in CIMP-H CRCs (*n* = 258) and of hypermethylated genes found in HUB040 and NC (*n* = 1,751) in HUB040 and NC vs. BC1 and BC2. (C and D) Gene set enrichment analysis with (C) Gene Ontology (GO) genesets and (D) genesets curated in MSigDB of overlapping genes from Hypermethylated genes in HUB040 and NC and Hypermethylated genes in CIMP-H CRC. Unpaired t test ∗∗∗∗*p* < 0.0001. CIMP, CpG island methylator phenotype; CRC, colorectal cancer; FDR, false discovery rate.

We next investigated if the CIMP phenotype was altered after correction of the BRAF^V600E^ mutation. To this end, we compared the methylation status of a previously identified set of 258 genes known to be hypermethylated in 90% of CIMP-high (H) colorectal cancers,^3^ in HUB040 and NC vs. BC1 and BC2. Strikingly, 53% (136/258) of the hypermethylated genes in CIMP-H CRC were hypermethylated in HUB040 and NC (Figure 2A). Moreover, RNA-seq analysis revealed that the expression of hypermethylated genes associated with both CIMP-H CRC and those identified in HUB040 and NC was strongly and significantly upregulated following correction of the BRAF*-*V600E mutation (Figure 2B). The overlapping gene set (i.e., demethylated and de-repressed following mutation correction; 136 genes) was significantly enriched with genes involved in developmental processes, RNA transcription regulation, and PRC2 target genes (Figures 2C and 2D).

### BRAF^V600E^ maintains DNA methylation of PRC2 target genes

**Figure 3:**
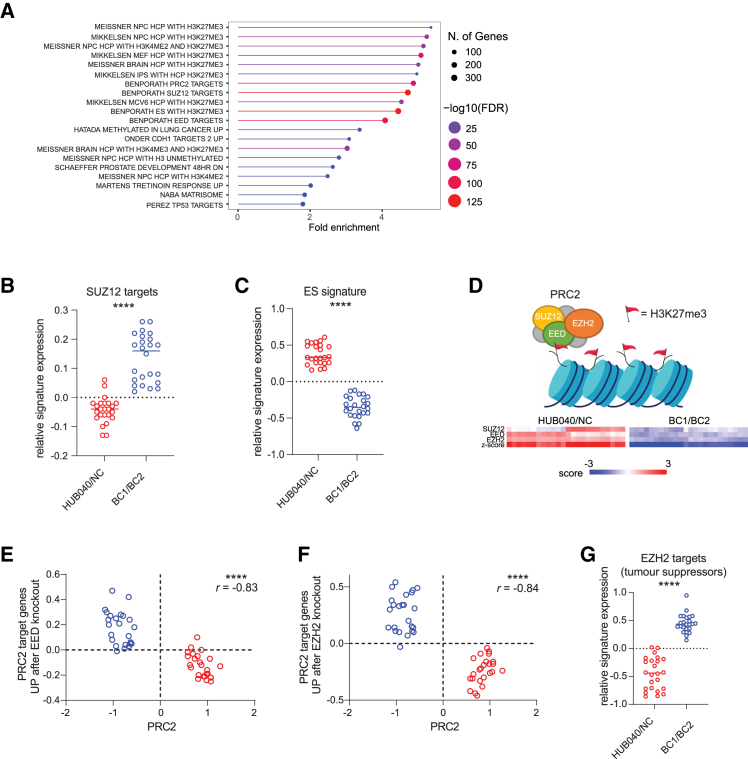
BRAF^V600E^ maintains DNA methylation of PRC2 target genes leading to suppression of PRC2 target gene transcription (A) Gene-set analysis of hypermethylated CpG-annotated genes in HUB040 and NC, using gene sets curated in MSigDB (shinyGO platform), sorted on fold enrichment. (B and C) Relative signature expression of SUZ12 targets (B) and (C) ES signature in HUB040 and NC vs. BC1 and BC2. (D) Expression of PRC2 core components SUZ12, EED, and EZH2 in HUB040 and NC vs. BC1 and BC2. (E and F) Correlation of gene signatures (genes UP after KO of PRC2 core components EED or EZH2) with PRC2 core component genes in HUB040 and NC vs. BC1 and BC2. Pearson’s correlation score is shown. (G) Relative signature expression of EZH2 targets (tumor suppressors) in HUB040 and NC vs. BC1 and BC2. Unpaired t test ∗∗∗∗*p* < 0.0001. ES, embryonic stem cell; FDR, false discovery rate; PRC2, polycomb repressor complex 2.

The hypermethylated CpGs in HUB040 and NC were annotated to 1751 genes. Gene ontology analysis using curated gene sets from the Molecular Signature Database (MSigDB^25^) on the ShinyGO^26^ platform, revealed a significant enrichment of several GO terms related to PRC2 activity (Figure 3A and Table S2). Hypermethylated genes in CIMP-H tumors show a strong overlap with PRC2 target genes^3^ and were also found to be transcriptionally upregulated following correction of the V600E mutation (Figure 2B). Moreover, our data reveal that PRC2 target genes, regardless of their association with the CIMP-high gene set, were methylated at significantly higher levels in HUB040 and NC vs. BC1 and BC2.

### Upregulation of PRC2 core components and downregulation of PRC2 target gene transcription in BRAF-V600E mutant organoids

Next, the effect of DNA methylation changes on gene expression was assessed. Our analysis revealed that of the 1,751 hypermethylated genes (found in HUB040 and NC), 413 genes (24%) were transcriptionally downregulated (*p* < 0.01) in HUB040 and NC vs. BC1 and BC2 (Figure S4A). Of the 450 hypomethylated genes (found in HUB040 and NC), 126 genes (28%) displayed transcriptional upregulation (*p* < 0.01) in HUB040 and NC vs. BC1 and BC2 (Figure S4B).

PRC2 activity related gene sets were significantly enriched in the gene set analysis of hypermethylated CpG annotated genes (found in HUB040 and NC) (Figure 3A). SUZ12 target genes were significantly repressed in HUB040 and NC (Figure 3B). PRC2 target gene repression is associated with an ES cell phenotype.^11^ Indeed, we found that the observed increase in SUZ12 target gene expression following correction of V600E, was paralleled by strong and significant suppression of an ES cell signature^11^ (Figure 3C).

Cancers with high expression of the ES signature often show high expression of the PRC2 core components EZH2 and EED.^11^ A marked loss of expression of three core components of PRC2 (SUZ12, EED, and EZH2) was observed following BRAF^V600E^ correction (Figure 3D) and of defined sets of PRC2 target genes (i.e., methylated genes showing increased expression after knockout of either EZH2 or EED^27^) (Figures 3E and 3F).

Moreover, overexpression of PRC2 core components, especially of the catalytic subunit EZH2, is observed in different types of cancers and is associated with poor prognosis.^28^ PRC2 can also promote carcinogenesis by EZH2-mediated silencing of tumor suppressor genes.^29^ Notably, we found that tumor suppressor genes, previously described to be occupied by EZH2, and concomitantly repressed, were also repressed in HUB040 and NC vs. BC1 and BC2 (Figure 3G).

To determine whether these changes were due to reduced MAPK activity in BRAF-corrected organoids, HUB040 organoids were treated with the BRAF inhibitor encorafenib, followed by RNA-seq analysis. This revealed a significant downregulation of the ES cell signature, as well as the PRC2 core components EZH2 and EED (Figure S5). However, the expression levels of SUZ12 target genes and EZH2 target genes remained unaffected.

Next to increased hypermethylation of PRC2 target genes our data also indicate higher PRC2 activity in HUB040 and NC. Therefore, we used the CUT&RUN technique to map H3K27me3 promoter occupancy in HUB040 and NC vs. BC1 and BC2. This revealed a strong relative increase of H3K27me3 occupancy at promoter regions in HUB040 and NC vs. BC1 and BC2 (Figure S6).

### Introduction of the BRAF-V600E mutation in healthy colon organoids induces hypermethylation of PRC2 target genes

To further explore the epigenetic impact of BRAF^V600E^, we analyzed two additional organoid models: a paired healthy colon organoid line derived from the same patient (HUB040-N) and a line where the V600E mutation was introduced into healthy colon organoids (HUB040-N-B).^21^ Genome-wide DNA methylation analysis revealed that hypermethylated regions occurred at higher frequency in HUB040-N-B organoids than in HUB040-N organoids, with a hypermethylation profile that began to resemble that of the BRAF-V600E mutant HUB040 line (Figure S7A). Conversely, the hypermethylation patterns of BRAF-corrected organoids (BC1 and BC2) aligned more closely with those of HUB040-N, suggesting reversal of BRAF^V600E^-driven methylation changes. In the HUB040-N-B organoid line, 1,035 genes (FDR <0.01, |Δβ| >0.2) were identified as hypermethylated relative to HUB040-N (Table S3). Gene ontology analysis of these hypermethylated genes revealed a strong association with PRC2 target genes, reinforcing the link between BRAF^V600E^ and PRC2-related epigenetic regulation (Figure S7B).

### DNA demethylation of BRAF^V600E^ organoids affects CIMP genes and PRC2 target gene expression

**Figure 4:**
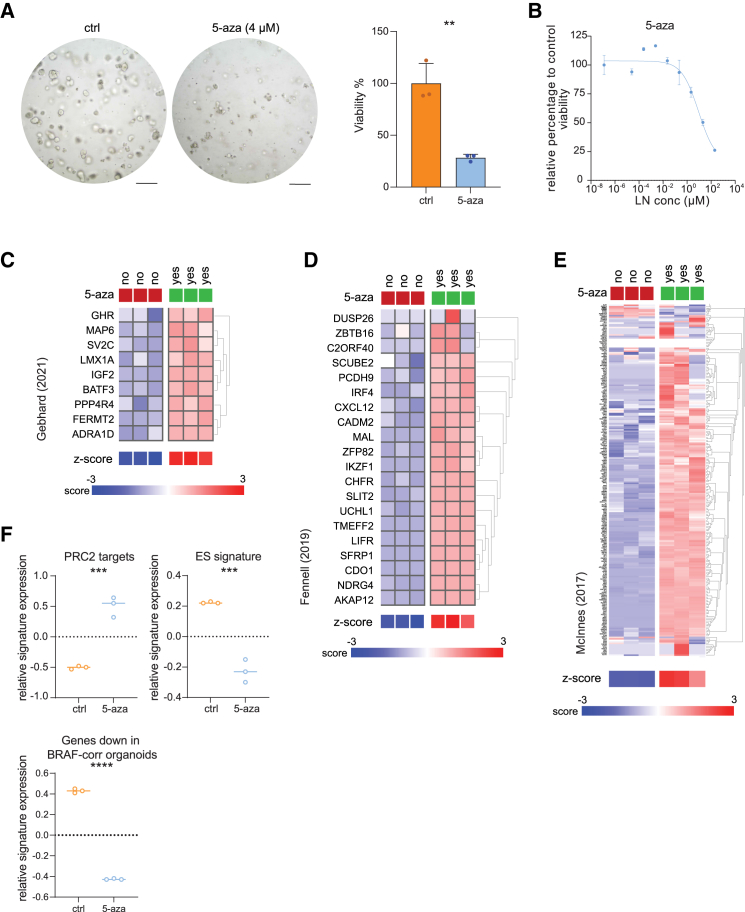
DNA demethylation of BRAF^V600E^ organoids affects CIMP genes and PRC2 target gene expression (A) HUB040 organoids treated with 4 μM 5-aza-2′-deoxycytidine (5-aza) for 10 days. Viability of organoids was measured with CellTiter-Glo 3D assays. Average of 3 technical replicates. Data are presented as mean ± SD. Scale bars, 200 μM. (B) Drug response curve of 5-aza treated HUB040 organoids. Organoids were treated for 7 days with indicated drug concentrations, after which viability was measured with CellTiter-Glo 3D assays. (C–E) CIMP marker gene expression (C) Gebhard et al. (IJC, 2021), (D) Fennell et al. (CMGH, 2019), and (E) McInnes et al. (BMC Cancer, 2017) in 5-aza treated organoids. (F) Relative signature expression of PRC2 target genes (Ben-Porath, 2008) and the embryonic stem (ES) cell signature in HUB040 vs. HUB040 5-aza treated organoids. Unpaired t test ∗∗*p* < 0.01, ∗∗∗*p* < 0.001, and ∗∗∗∗*p* < 0.0001. ES, embryonic stem cell; PRC2, polycomb repressor complex 2.

The aforementioned data suggest that DNA methylation may be an important driver of the transformed phenotype in BRAF^V600E^-mutant colon cancer. To test this, we used the DNA demethylating agent 5-aza-2′-deoxycytidine (5-aza) and tested its effect on the growth properties and the transcriptome of HUB040. 5-aza treatment strongly reduced organoid-forming capacity (Figures 4A and 4B). Transcriptome analysis revealed that 5-aza caused a strong and significant increase in the expression of several CIMP marker gene panels^3^^,^^19^^,^^30^ (Figures 4C–4E). Moreover, PRC2 target genes were also upregulated (de-repressed) in 5-aza-treated organoids, and this was paralleled by strong and significantly decreased expression of the ES cell signature (Figures 4F and S8). This aligns with the observation that genes downregulated in BRAF-corrected organoids also showed reduced expression in 5-aza treated organoids (Figure 4F), further supporting the similarities between 5-aza treatment and BRAF-V600E correction with respect to regulation of CIMP, PRC2 target genes, and an ES-like phenotype.

### DNA demethylation treatment of BRAF^V600E^ organoids results in durable treatment responses

As 5-aza treatment resulted in upregulation of CIMP and PRC2 target genes, accompanied by a decrease of the aggressive ES-like phenotype, we tested its treatment potential in 4 BRAF^V600E^-mutant CRC PDOs. PDOs were exposed to 5-aza for 10 days after which cell viability was measured and regrowth was assessed over the time span of a week following drug wash-out. Out of 4 PDOs tested, 3 PDOs were highly sensitive to 5-aza treatment (Figure S9A). Interestingly, a durable treatment response was observed in all 4 PDOs as prolonged culturing in CRC growth medium after 5-aza wash-out did not lead to re-growth of treated PDOs (Figure S9B). Additionally, combination treatment of 5-aza and PRC2 inhibitors (GSK-126 and EED226), showed a significant synergistic effect (Figure S10), in line with previous research.^31^

## Discussion

In this study, we have shown that the BRAF^V600E^ mutation plays a major role in maintenance of epigenetic changes that are associated with tumor aggressiveness. We have found that correction of the V600E mutation in BRAF results in demethylation of CIMP genes and of PRC2 target genes in colon cancer organoids.

First, more than half of the genes that are hypermethylated in CIMP-H colorectal cancers^3^ showed a significant loss of DNA methylation following correction of V600E, leading to de-repression of gene expression. However, while BRAF plays a significant role in maintaining the CIMP-H state, it is likely not the sole contributor, as other factors—including age-related epigenetic changes^16^^,^^17^^,^^18^—may also influence the methylation landscape. Furthermore, as gene regulation is multifactorial, BRAF^V600E^ may impact chromatin remodeling or histone modifications, which could explain why some genes retained methylation after BRAF correction. Previously, it has been questioned whether CIMP is a direct consequence of the BRAF^V600E^ mutation, or whether CIMP generates a cell state that is conducive to cellular transformation by BRAF^V600E.^^14^^,^^17^^,^^18^ Our data unequivocally demonstrate that the V600E mutation in BRAF is essential to maintain the CIMP-H phenotype and global CIMP-H-associated gene repression, and that loss of DNA methylation results in loss of the transformed phenotype. Noteworthy, the PDO model that was used in this model also harbored a pathogenic cancer gene census mutation in *ARID1A* (p.Q473∗), a component of the SWI/SNF chromatin remodeling complex. Loss-of-function mutations in *ARID1A* are potential drivers of the CIMP phenotype.^32^ Clearly, in the model applied here the *ARID1A* mutation was not sufficient to maintain CIMP in the absence of the BRAF^V600E^ mutation.

Secondly, we showed that BRAF^V600E^ mutation correction leads to demethylation of PRC2 target genes. Silencing of PRC2 targets contributes to an embryonic stem cell-like state associated with poorly differentiated tumors by repressing important developmental regulators, including tumor suppressor genes.^11^ Aging is thought to be the primary factor accelerating hypermethylation changes in genes that play a role in development and differentiation.^17^^,^^33^^,^^34^ However, our results suggest that BRAF^V600E^ plays a significant role in the maintenance of hypermethylation of PRC2 target genes, including a set of tumor suppressor genes. Furthermore, expression of the core genes constituting the PRC2 complex itself was significantly reduced following BRAF^V600E^ mutation correction, which was paralleled by upregulation of PRC2 target genes. We did not find evidence of differential DNA methylation at the promoters/gene bodies of PRC2 complex genes. It is plausible that BRAF^V600E^ could influence the activity of transcription factors or chromatin remodelers, potentially impacting the accessibility of these loci, resulting in upregulation of these genes. In addition to the upregulation of PRC2 core components, significant increases in UHRF1, HDAC2, MBD3, and MBD4 expression were also observed in BRAF-V600E mutant organoids, highlighting the coordinated involvement of multiple epigenetic regulators. Analysis of larger colorectal cancer datasets further revealed consistent upregulation of EZH2, EED, and UHRF1 in BRAF-mutant CRCs compared to BRAF-wildtype tumors, reinforcing the broader relevance of these findings. Collectively, the increased expression of these key epigenetic modifiers could potentially drive the epigenetic reprogramming observed in BRAF-V600E mutant organoids, although our work does not provide direct mechanistic evidence for this. Importantly, no changes in the expression of DNMTs or TETs were detected, indicating that BRAF^V600E^ maintains hypermethylation by modulating the expression of other epigenetic regulators rather than directly altering DNMT or TET expression. We propose therefore, that DNA methylation and PRC2-mediated histone methylation cooperatively alter the epigenetic landscape in BRAF^V600E^ mutant CRCs, and that this generates an aggressive embryonic-like tumor phenotype.

BRAF^V600E^-mutant tumors represent an aggressive hard-to-treat entity within colorectal cancer.^35^^,^^36^ Currently, the most effective treatment constitutes combined inhibition of BRAF, MEK, and EGFR.^37^^,^^38^ However, even with this combination of targeted drugs, the median overall survival in this patient group is only 9 months. Clearly, more effective treatment strategies remain urgently needed. Our study suggests that epigenetic therapies may represent an alternative treatment strategy for BRAF^V600E^-mutant colon cancer. PRC2 inhibitors are currently tested in clinical trials for several cancer types,^39^ and decitabine (5-aza-2′-deoxycytidine) is extensively used in the treatment of myelodysplastic syndromes and acute myeloid leukemia.^40^ Although a recent proof-of-concept study in colon cancer reported limited success in achieving DNA demethylation with decitabine,^41^ our results highlight the need for further investigation into optimizing epigenetic therapies for BRAF^V600E^-driven cancers. By exploring such approaches, we may identify new treatment options for patients with BRAF-V600E mutant colon cancer.

### Limitations of the study

While this study provides significant insights into the epigenetic regulation associated with the BRAF-V600E mutation in colorectal cancer, several limitations warrant consideration.

First, our findings stem from experiments utilizing a PDO model generated from a single advanced-stage colon tumor. Given the considerable genetic and molecular diversity among BRAF-V600E mutant colorectal cancers, these results may not fully capture the variability present in broader clinical populations. Second, although we observe differential expression of key epigenetic modifiers between BRAF-mutant and BRAF-corrected CRC organoids and in BRAF-mutant versus BRAF-wildtype CRC tumors, these changes are modest in magnitude. Additionally, we observed transcriptional variation between BRAF-V600E mutant isogenic lines (HUB040 versus NC), complicating mechanistic interpretation. As such, while our data indicate that the BRAF-V600E mutation influences the epigenetic landscape and that altered expression of specific chromatin regulators may contribute to this, they do not definitively establish that this is the underlying mechanism. Third, the study focuses on the BRAF-V600E mutation and its effects on DNA methylation and PRC2 target gene regulation, but other mutations (e.g., *ARID1A*) present in the PDOs may also contribute to the observed epigenetic landscape. The interplay between these mutations remains unclear. Additionally, while the study highlights the therapeutic potential of epigenetic modifiers like decitabine and PRC2 inhibitors, the durability and specificity of these treatments in clinical settings require further validation. The inability of decitabine to achieve DNA demethylation in prior studies also underscores the need for optimization of treatment protocols. Lastly, the lack of direct evidence linking BRAF^V600E^ to transcription factor or chromatin remodeler activity leaves a gap in understanding the precise mechanisms driving PRC2 component upregulation. Further research is needed to clarify these mechanisms, potentially opening new avenues for developing effective strategies against this aggressive form of colorectal cancer.

## Resource availability

### Lead contact

Further information and requests for resources and reagents should be directed to and will be fulfilled by the lead contact, Onno Kranenburg (o.kranenburg@umcutrecht.nl).

### Materials availability

Plasmids generated in this study are available from the lead contact. PDOs may be obtained from the lead contact with a completed materials transfer agreement.

### Data and code availability

• The Infinium MethylationEPIC BeadChip data generated in this study have been deposited in ArrayExpress, accessions E-MTAB-13283 (BRAF-V600E correction) and E-MTAB-13290 (BRAF-V600E knock-in). The RNA-seq data generated in this study have been deposited in ArrayExpress, accessions E-MTAB-13806 (BRAF-V600E correction). RNA-seq data are also accessible under request in the “R2: Genomics Analysis and Visualization Platform (http://r2.amc.nl)”.
• This paper does not report original code.
• Any additional information required to reanalyze the data reported in this work paper is available from the lead contact upon request.

## Acknowledgments

The authors thank the Pathology department at the UMC Utrecht for running the Infinium MethylationEPIC array. We acknowledge the Utrecht Sequencing Facility (USEQ) for providing RNA sequencing service and data. This work was supported by the Dutch Foundation for medical scientific research ZonMW TOP Grant 91218050 (to M.M.M., O.K., and H.J.G.S.). M.M.M. is funded by Oncode Institute, which is partly financed by the Dutch Cancer Society (KWF), KWF Grant 13112, TKI/KWF grant 14853, and the NWO Gravitation project IMAGINE!.

## Author contributions

L.E.B., J.M.B., H.J.G.S., M.M.M., and O.K. conceptualized the project. L.E.B. and O.K. designed and analyzed experiments. J.M.B., E.K., A.V., J.B.P., and N.F. provided methodological expertise. I.H.M.B.R., H.J.G.S., M.M.M., and O.K. provided supervision for this study. L.E.B. and O.K. wrote the manuscript. All authors reviewed and edited the final version of the manuscript.

## Declaration of interests

The authors declare no competing interests.

## STAR★Methods

### Key resources table

**Table:** 

REAGENT or RESOURCE	SOURCE	IDENTIFIER
**Biological samples**		

Patient-Derived Organoids (PDOs) – CRC and healthy colon organoids	Hubrecht Organoid Technology	HUB-Cancer TcBio#12–09

**Chemicals, peptides, and recombinant proteins**		

Decitabine (5-Aza-2′-deoxycytidine)	AbMole	M2052
Dispase II	Roche	12273600
TrypLE™ Express Enzyme	ThermoFisher Scientific	12604013
Matrigel	Corning	354234
Advanced DMEM/F12	ThermoFisher Scientific	12634010
B27 supplement	ThermoFisher Scientific	17504044
N-acetyl-L-cysteine	Sigma-Aldrich	A9165
Nicotinamide	Sigma-Aldrich	N0636
A83-10	Tocris	2939
SB202190	Sigma-Aldrich	S7067
EGF (Recombinant Human EGF)	PeproTech	AF-100-15
Glutamax	ThermoFisher Scientific	35050–038
HEPES	ThermoFisher Scientific	15630–056
Penicillin/Streptomycin	Lonza	DE17-602E
Noggin conditioned medium	Prepared in house	N/A
ROCK inhibitor (Y-26732)	Sigma-Aldrich	129830-38-2

**Critical commercial assays**		

QIAamp DNA Micro Kit	Qiagen	56304
CellTiter-Glo® 3D Cell Viability Assay	Promega	G9681
RNeasy® Mini Kit	Qiagen	74004
Infinium Human MethylationEPIC Beadchip	Illumina	20087706

**Deposited data**		

MethylationEPIC data - BRAF-V600E correction	ArrayExpress	E-MTAB-13283
RNAseq data - BRAF-V600E correction	ArrayExpress	E-MTAB-13806
RNAseq data - BRAF-V600E knock-in	ArrayExpress	E-MTAB-13920

**Oligonucleotides**		

gRNA-BRAF-V600E correction-hLbCpf1 (DR) sequence (5′-AATTTCTACTAAGTGTAGAT-3′) guide sequence (5′-GTCTAGCTACAGAGAAATCTCGA-3′)	This paper	N/A
Pair1_BRAF-exon 15_forward 5′-GGAGAGCAGGATACCACAGC-3′	This paper	N/A
Pair1_BRAF-exon 15_reverse 5′-CCACCGGTAGGCGCCAAC-3′	This paper	N/A
Pair2_BRAF-exon 15_forward 5′-CCTCTGACCTTGCTCAGTGG-3′	This paper	N/A
Pair2_BRAF-exon 15_reverse 5′-TGGATCCAGACAACTGTTCAA-3′	This paper	N/A

**Recombinant DNA**		

phU6-gRNA	Addgene	53188
pcDNA3.1-hLbCpf1	Addgene	69988
BRAF-V600E_correction template	This paper	N/A
hCas9	Addgene	41815

**Software and algorithms**		

shinyÉPICo	https://bioconductor.org Morante-Palacios and Ballestar^41^	N/A
R (version 4.2.1)	https://cran.r-project.org	N/A
GraphPad Prism 9	GraphPad software	N/A
R2 Genomics Analysis and Visualization Platform	R2; http://r2.amc.nl	N/A

### Experimental model and study participant details

#### CRC/healthy colon patient-derived organoid cultures

The collection and processing of human colorectal cancer and normal colon tissues (HUB-Cancer TCBio protocol ID number 12–093) was approved by the Biobank Research Ethics Committee of the University Medical Center Utrecht (Utrecht, The Netherlands). Written informed consent from the donors for research use of tissue in this study was obtained prior to the acquisition of the specimen according to the principles expressed in the Declaration of Helsinki.

The CRC patient-derived organoid line with identifier HUB-02-B2-040 (CRC) was previously established and characterized.^42^ The organoids utilized in this study originated from a 51-year-old female stage IV colon cancer patient with peritoneal metastases. The tumor was classified as microsatellite stable (MSS) and MLH1 protein staining was normal. Human colon cancer organoids were cultured as described previously.^43^ The organoids were embedded in Matrigel (Corning) and cultured with advanced DMEM/F12 medium (Gibco) with 1x Penicillin/Streptomycin (Lonza), 10 mM HEPES (Invitrogen) and 2 mM Glutamax (Gibco). The basal medium was further supplemented with 1% Noggin conditioned medium, 1x B27 Supplement (Gibco), 10 mM Nicotinamide (Sigma-Aldrich), 1.25 mM N-acetylcysteine (Sigma-Aldrich), 500 nM A83-01 (Tocris), and 10 μM SB202190 (Sigma-Aldrich). After organoid transfection the growth medium of BRAF^V600E^ and BRAF^E600V^ organoids was supplemented with 50 ng/mL EGF (PreproTech). Organoids were split every week through TrypLE Express (Gibco) treatment. Culture medium after splitting was supplemented with 10 μM Y-27632 dihydrochloride (Sigma-Aldrich). For selection of BRAF^E600V^ organoids 1 μg/μL puromycin was supplemented in the growth medium. The absence of mycoplasma contamination was routinely confirmed using a PCR-based assay.

### Method details

#### Gene engineering of organoids and genotyping

To correct the BRAF^V600E^ mutation in the HUB040 CRC PDO, an estimated 1 x 10^6^ cells of this PDO were electroporated with the single-guide RNA plasmid, pcDNA3.1-hLbCpf1 (Addgene, 69988) and a BRAF-V600E correction template plasmid. To specifically target the mutant allele, a guide RNA was designed to solely bind the mutant sequence.^44^ The gRNA for hLbCpf1 is composed of the 20-nt direct repeat (DR) sequence (5′-AATTTCTACTAAGTGTAGAT-3′) followed by a 23-nt guide sequence (5′-GTCTAGCTACAGAGAAATCTCGA-3′). Off-target effects of the guide sequence were predicted using the Cas-OFFinder online tool.^45^ All potential off-targets up to 4 mismatches were taken into account. Oligonucleotide duplexes corresponding to spacer sequences were annealed and ligated into BbsI-digested phU6-gRNA (Addgene, 53188). For the generation of the BRAF-V600E correction template site-directed mutagenesis was performed on a BRAF-V600E mutation template that was generated earlier.^46^ Clonal lines were established under puromycin selection, in which single cells were grown into single organoid structures that were individually picked and expanded. Genomic DNA was extracted from expanded single organoids using the QIAamp DNA Micro Kit (Qiagen) and correction of the BRAF^V600E^ mutation was confirmed by Sanger sequencing and the ddPCR BRAF V600 Screening Kit (Bio-Rad). The sequences of primers used for Sanger sequencing are: Pair 1 (5′-3′) Fw: GGAGAGCAGGATACCACAGC and Rv: CCACCGGTAGGCGCCAAC, and Pair 2 (5′-3′) Fw: CCTCTGACCTTGCTCAGTGG and Rv: TGGATCCAGACAACTGTTCAA. For generation of HUB040-N-B organoids an estimated 1 x 10^6^ cells of HUB040-N organoids were electroporated with the single-guide RNA plasmid (5′-GAAGACCTCACAGTAAAAAT-3′) ligated into BbsI-digested phU6-gRNA (Addgene, 53188), a human codon-optimized Cas9 expression plasmid (Addgene, 41815), and the BRAF-V600E mutation template.^46^ Clonal lines were established under puromycin selection and introduction of the BRAF-V600E mutation was confirmed by Sanger sequencing using Primer pair 2.

#### Infinium MethylationEPIC BeadChip and data analysis

Genomic DNA from BRAF^V600E^ organoids (HUB040/NC) and BRAF^E600V^ organoids (BC1/BC2), and HUB040-N and HUB040-N-B organoids was isolated using the QIAamp DNA Micro Kit (Qiagen). Bisulfite-converted DNA was amplified, fragmented, and hybridized to Illumina Infinium Human MethylationEPIC Beadchip using standard Illumina protocol. The raw intensity files (idat) generated by the EPIC arrays were imported into the R programming environment (v4.3.0) using RStudio (2023.03.0). MethylationEPIC analysis was performed using the shinyÉPICo application.^47^ Using the *minfi*^48^ package within the application, array normalization was performed against parameters provided by Illumina, and non-CpG probes and probes with SNPs were excluded. Differentially methylated CpGs were detected using the *limma* package.^49^ The DNA methylation level for each CpG site is reported as a beta value, ranging from 0 (not methylated) to 1 (fully methylated). To identify differentially methylated CpGs, the methylation difference per CpG was calculated as the mean beta value of BRAF^V600E^ organoids (HUB040/NC) minus the mean beta value of BRAF^E600V^ organoids (BC1/BC2) or as the mean beta value of HUB040-N-B organoids minus the mean beta value of HUB040-N organoids. Those with a methylation difference |< 0.2| were filtered out, as well as those with an FDR > 0.05. A CpG is considered “hypermethylated” in BRAF^V600E^ organoids (HUB040/NC or HUB040-N-B) when the methylation difference is >0.2 and as “hypomethylated” when the methylation difference is < -0.2. Studies were focused on CpGs in CpG Island (CGI) regions. The overlap with CGI and the annotated gene was performed using the CGI track from the UCSC genome browser, and Refseq gene annotations based on the GRCh37/hg19 human reference. ShinyGO^26^ was employed to complete gene ontology (GO) analysis. Gene set analysis was performed against all gene sets and signatures curated in MSigDB.^25^

#### RNA sequencing and data analysis

Total RNA was extracted from BRAF^V600E^ (HUB040/NC) and BRAF^E600V^ organoids (BC1/BC2), and from HUB040 (ctrl, 7 days old) and HUB040 5-aza-2′-deoxycytidine treated organoids (7 days treated with 4 μM decitabine).

Organoids were collected from Matrigel by enzymatic digestion using 1 mg/mL dispase (Gibco) for 10 min at 37°C and were lysed in RLT-buffer (Qiagen) supplemented with 1% β-mercaptoethanol. Total RNA was isolated using the RNeasy Mini Kit (Qiagen) according to the manufacturer’s protocol. DNase (Qiagen) treatment was included to avoid possible genomic DNA contamination. Extracted RNA was quantified using a Qubit fluorometer and quality assessed using bioanalyzer quality control. Libraries were prepared using Truseq RNA stranded polyA (Illumina) according to the manufacturer’s directions. Sequencing was performed on a NextSeq2000 (Illumina; 1 × 50 bp) and base calling performed using RTA (Illumina). Quality control of raw reads was done using FastQC.

The RNAseq datasets were uploaded into R2 (http://r2.amc.nl) for subsequent bioinformatics analyses and are available on the platform. Differential gene expression analysis was performed with the DESeq2 package^50^ within the R2 environment, using a cut-off of *p* < 0.01 (ANOVA) with multiple testing correction by false discovery rate. Gene enrichment analysis was performed using ShinyGO.^26^

#### Organoid drug assays and regrowth assays

HUB040, HUB096, HUB098 and HUB006 organoids were dissociated into single cells using TrypLE Express (Gibco) and re-suspended in a mixture of CRC growth medium and Basement Membrane Extract (R&D Systems) (1:3). Droplets of this mixture (4 μL per well) were seeded in 2x 96-well plates and CRC growth medium was added to the wells. Decitabine (5-Aza-2′-deoxycytidine; AbMole) and/or PRC2 inhibitors (GSK126; Selleckchem and EED226; Selleckchem) was/were added to the wells using the TECAN D300e Digital Dispenser. CRC growth medium and drugs were refreshed every 3–4 days. Cell viability of 10 days treated organoids was measured with CellTiter-Glo 3D assays (Promega) in 1 plate. Luminescence levels were measured using a SpectraMax M5 microplate reader (Molecular Devices). In the other 96-wells plate drugs were washed out twice with basal medium and CRC growth medium was added to measure regrowth ability of 10-day treated organoids for another 7 days, with a medium re-fresh in between after 3 days. Re-growth ability was also measured with CellTiter-Glo 3D assays.

#### ChIC (H3K27me3) and data analysis

Bulk ChIC libraries were processed from BRAF^V600E^ organoids (HUB040/NC) and BRAF^E600V^ organoids (BC1/BC2). Raw reads from sc-sortChIC are demultiplexed using SingleCellMultiomics *demux.py* (v0.1.23). Then adapters are removed for all libraries. ChIC reads are mapped with *minimap2*^51^ (v1.15.1-gcdec39d) to assembly GRCh38.p13 and common contaminants (*Cutibacterium*, *E. coli K-12*, *E. coli* Lambda Phage, *E. coli RHB09-C15* and mitochondria). Next, molecules are deduplicated based on strand, MNase cut site location and UMI generating unique molecules for counting. Next, molecules are filtered using SingleCellMultiomics *bamtagmultiome.py*. Reads mapping to problematic genomic regions or contaminants are removed. Problematic genomic regions are identified using the ENCODE blacklist.^52^ Coverage tracks in BigWig format were generated using deepTools^53^ *bamCoverage* with 50bp bin-sized regions and normalized to CPM. Log-fold change tracks are calculated using *bedtools*^54^ bigwigCompare, with a bin-size of 10kb. For quality control, the amount of unique molecules are reported as percentage of duplicated molecules. Further, percentage of reads mapping to genes, TSS and TES are reported. TSS are defined as regions 5kb upstream of a gene body and TES as regions 5kbp downstream of a gene body. Coverage distribution for all libraries near TSS was calculated using SingleCellMultiOmics *scsortchicfeaturedensitytable.py*. All QC statistics and plots are reported in MultiQC.^55^

Log2-fold changes across conditions are calculated for cpm per genes (length normalized). Only genes with a length above 50kb are taken into account. Conditions that are compared are BRAF^V600E^ organoids (HUB040/NC) vs. BRAF^E600V^ organoids (BC1/BC2). Thresholds are applied to select most variable genes across the comparisons. A minimum log2-fold change of 1.1 is required.

### Quantification and statistical analysis

Statistical analyses were performed using Prism v9.4.0 software. Significances were determined using unpaired 2-tailed Student’s t test, and correlations (R) were measured as Pearson’s correlations, unless otherwise indicated. *p* < 0.05 was considered significant.

## Supplemental information

Document S1. Figures S1–S10

Table S1. Differentially methylated CpG sites in HUB040 and NC (vs. BC1 and BC2)

Table S2. Gene enrichment analysis of hypermethylated genes in HUB040 and NC

Table S3. Hypermethylated CpG sites (islands) in HUB040-N-B (vs. HUB040-N)
