# Supplementary material for: BRAFV600E maintains the CpG island methylator phenotype, and DNA methylation of PRC2 targets genes in colon cancer
Source: iScience. 2025 Jun 14;28(7):112905. doi: 10.1016/j.isci.2025.112905 (PMC12269043; doi:10.1016/j.isci.2025.112905)
Supplement: Document S1. Figures S1–S10 [file mmc1.pdf]

iScience, Volume *28*

Supplemental information

BRAF^{V600E} maintains the CpG island methylator phenotype, and DNA methylation of PRC2 targets genes in colon cancer

Layla El Bouazzaoui, Jeroen M. Bugter, Emre Küçükköse, André Verheem, Jasmin B. Post, Nicola Fenderico, Inne H.M. Borel Rinkes, Hugo J.G. Snippert, Madelon M. Maurice, and Onno Kranenburg

Supplementary figures

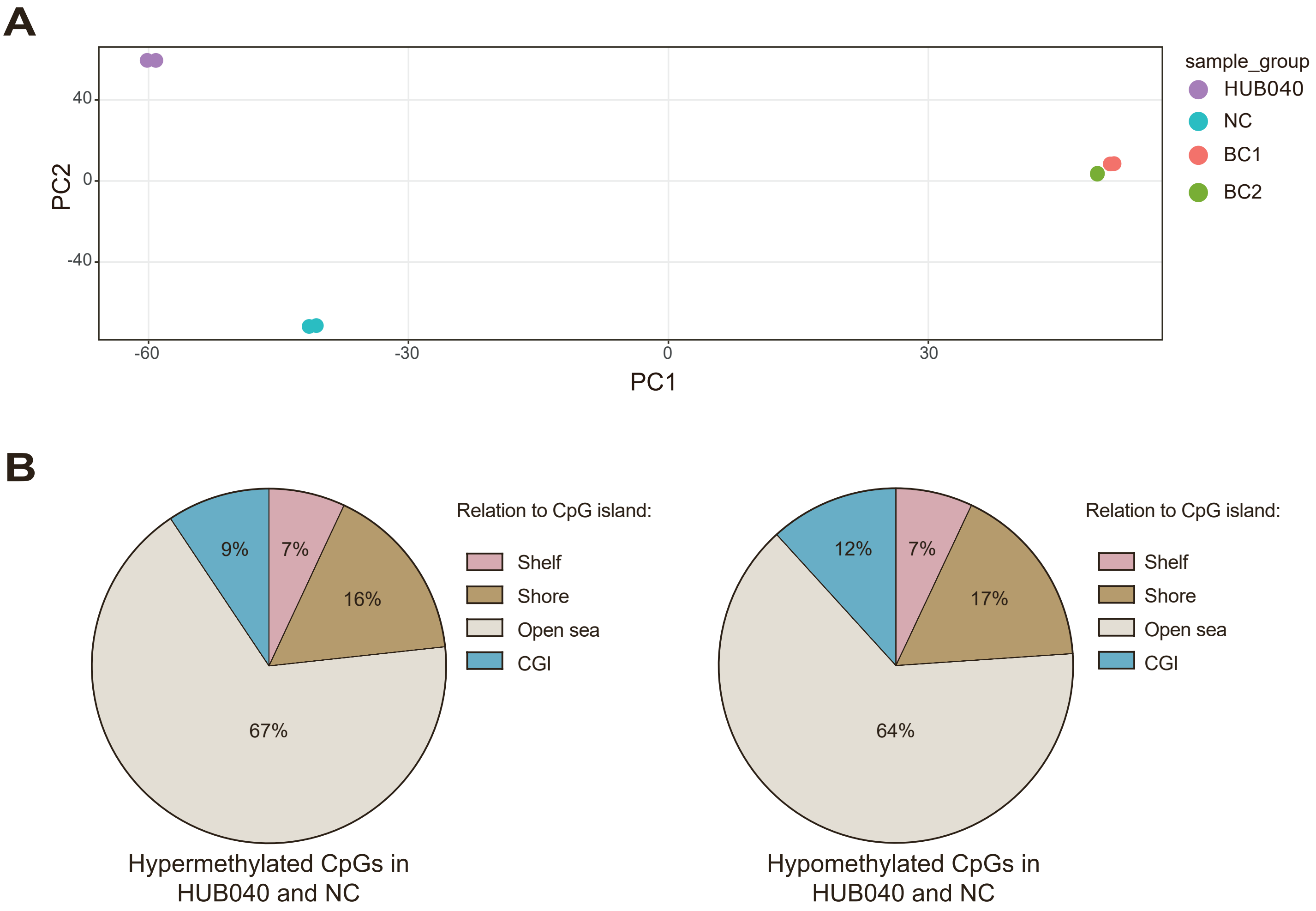

Figure S1: CpG methylation in BRAF^{V600E} and BRAF^{E600V} organoids. (A) PCA of whole DNA methylomes of BRAF^{V600E} (HUB040 and NC) and BRAF^{E600V} organoids (BC1 and BC2). Two technical replicates per PDO were generated. (B) Association of aberrantly methylated sites with CpG islands (shores are < 2 kb and shelves are 2–4 kb from a CpG island). CGI, CpG island.

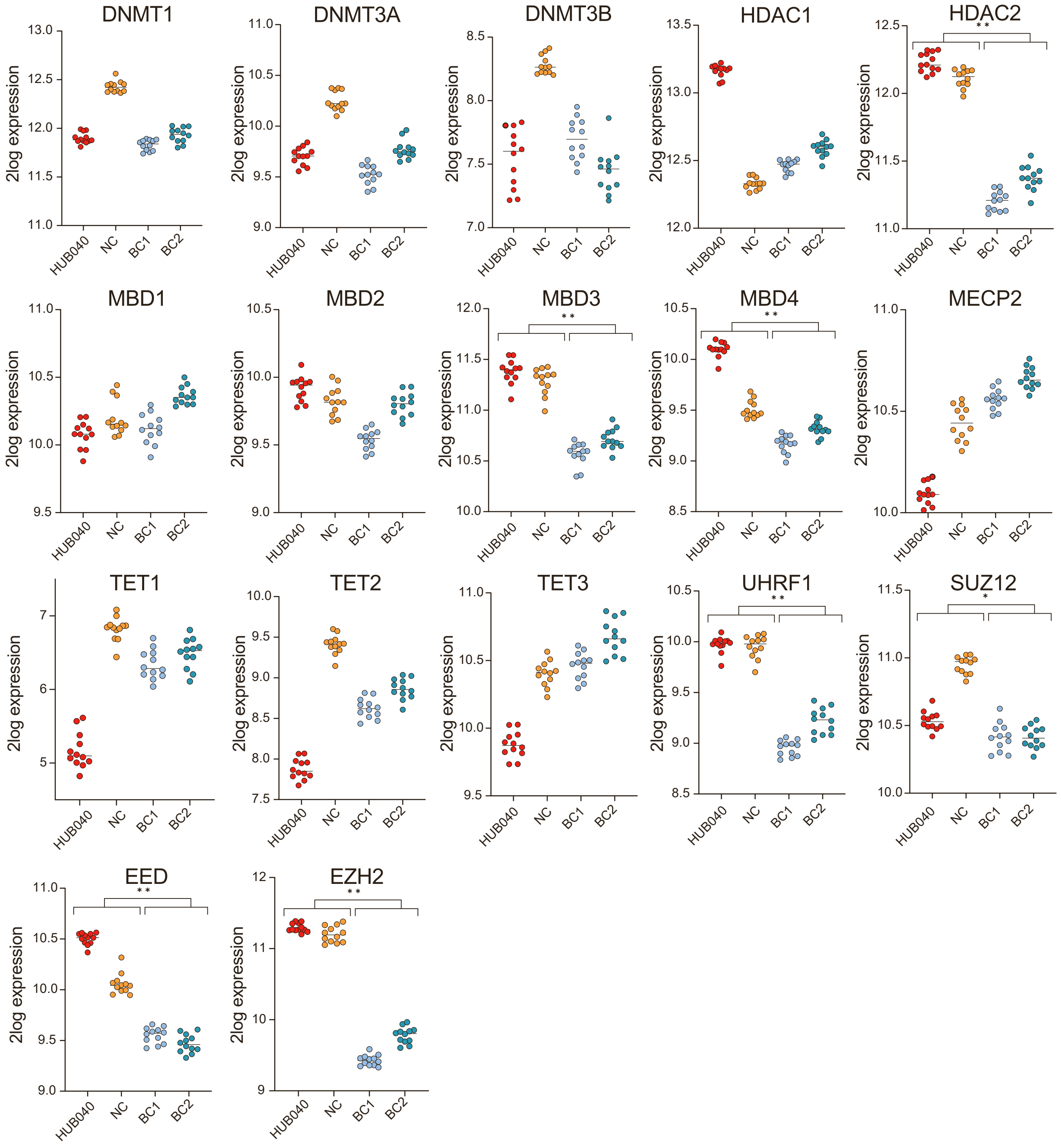

Figure S2: Expression of key epigenetic modifiers in BRAF^{V600E} and BRAF^{E600V} organoids. Differential expression (2log) between BRAF-mutant (HUB040, NC) and BRAF-corrected (BC1, BC2) organoids was assessed using paired Wilcoxon signed-rank tests for HUB040 vs BC1, HUB040 vs BC2, NC vs BC1, and NC vs BC2. p-values were Bonferroni-corrected, and genes were considered significant if corrected p-values were < 0.05 for all comparisons. *P < .05, **P < .01.

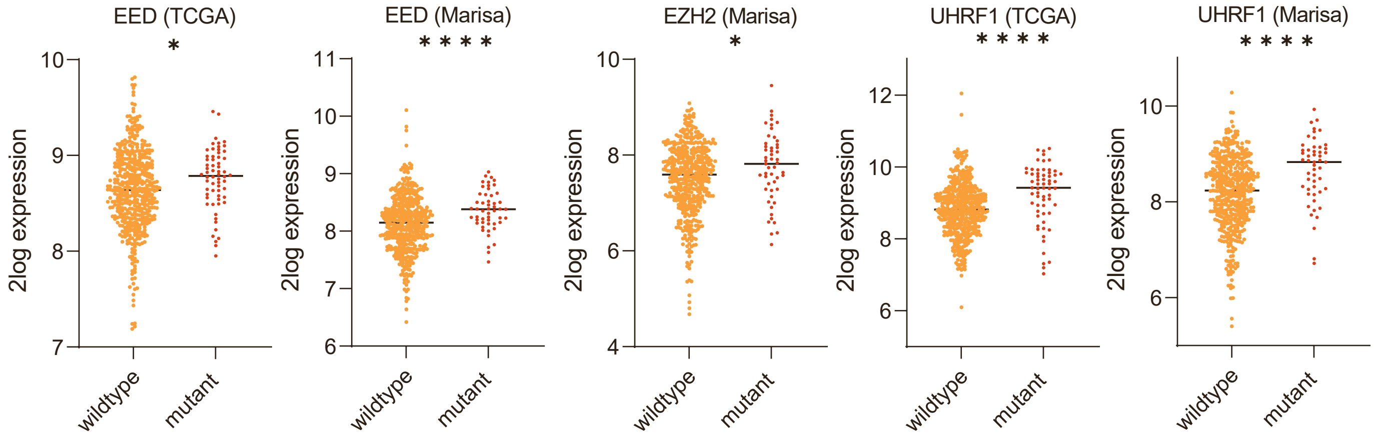

Figure S3: Expression of epigenetic modifiers in BRAF-mutant versus BRAF-wildtype CRCs. 2log expression of EED, EZH2, and UHRF1 in CRC TCGA dataset (BRAF-M, n=62; BRAF-wildtype n=470), and colon cancer dataset Marisa et al. (BRAF-M, n=51; BRAF-wildtype n=461). Unpaired t-test *P < 0.05, ****P < .0001. TCGA, The Cancer Genome Atlas.

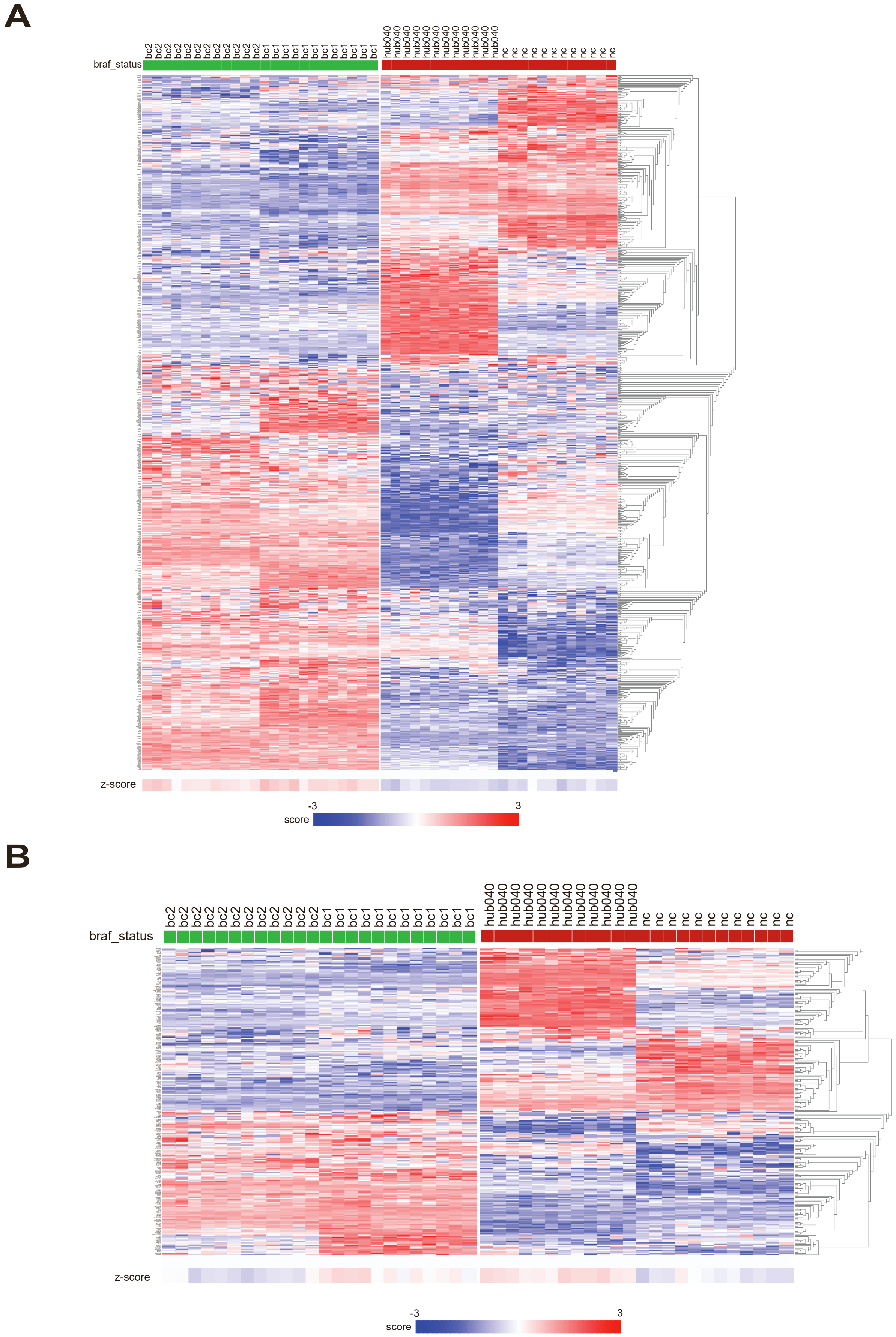

Figure S4: Expression of hypermethylated and hypomethylated genes. (A) Gene expression of hypermethylated CpG annotated genes (in HUB040 and NC) with a *P* value < 0.01 (n=711). 413 genes were concordantly transcriptionally repressed in HUB040 and NC. (B) Gene expression of hypomethylated CpG annotated genes (in HUB040 and NC) with a *P* value < 0.01 (n=236). 126 genes were concordantly transcriptionally upregulated in HUB040 and NC.

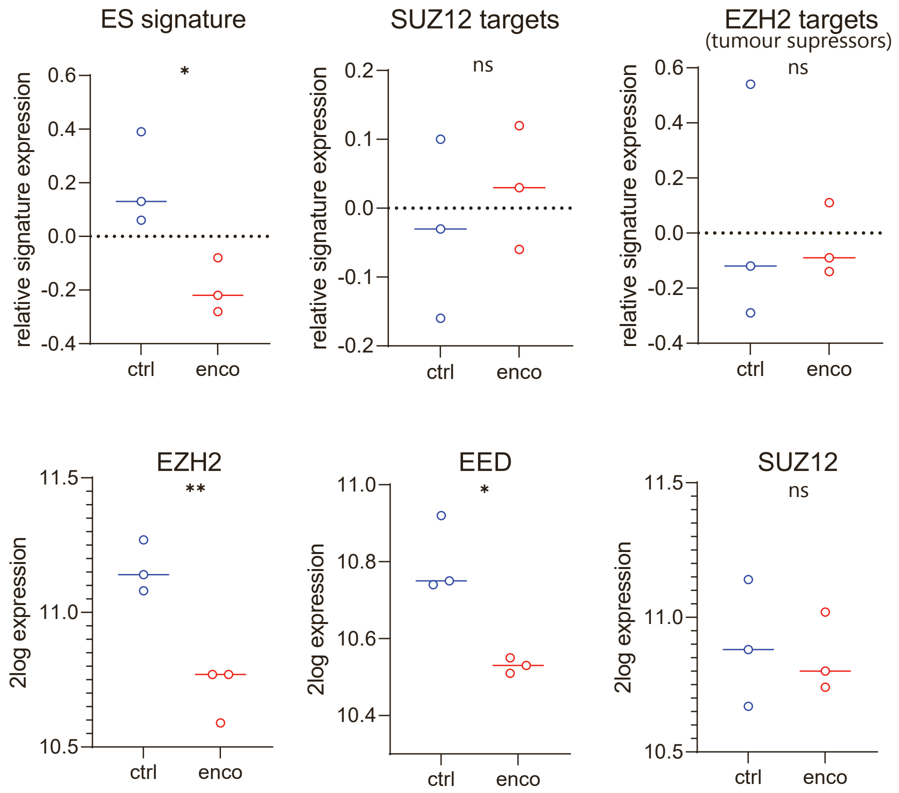

Figure S5: Embryonic stem cell signature and PRC2 core components expression in encorafenib treated BRAF^{V600E} organoids. Relative signature expression (z-scores) of the ES signature, SUZ12 targets, EZH2 targets, and the log2 expression of PRC2 core components EZH2, EED and SUZ12 in HUB040 organoids treated with 1 μ M encorafenib for 4 days. Three technical replicates per condition. Unpaired t-test *P < 0.05, **P < .01.

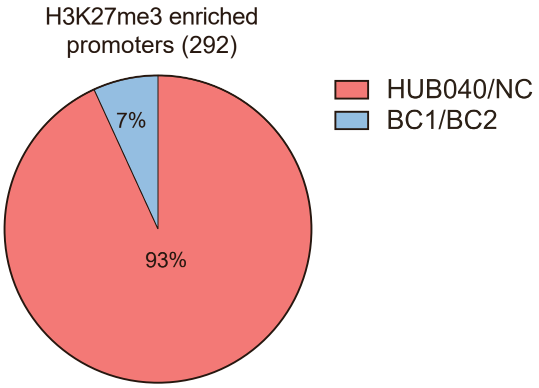

Figure S6: H3K27me3 promoter occupancy in $BRAF^{V600E}$ and $BRAF^{E600V}$ organoids. H3K27me3 enrichment analysis of gene promoters in HUB040 and NC *vs.* BC1 and BC2 (min. |log2FC| is 1.1).

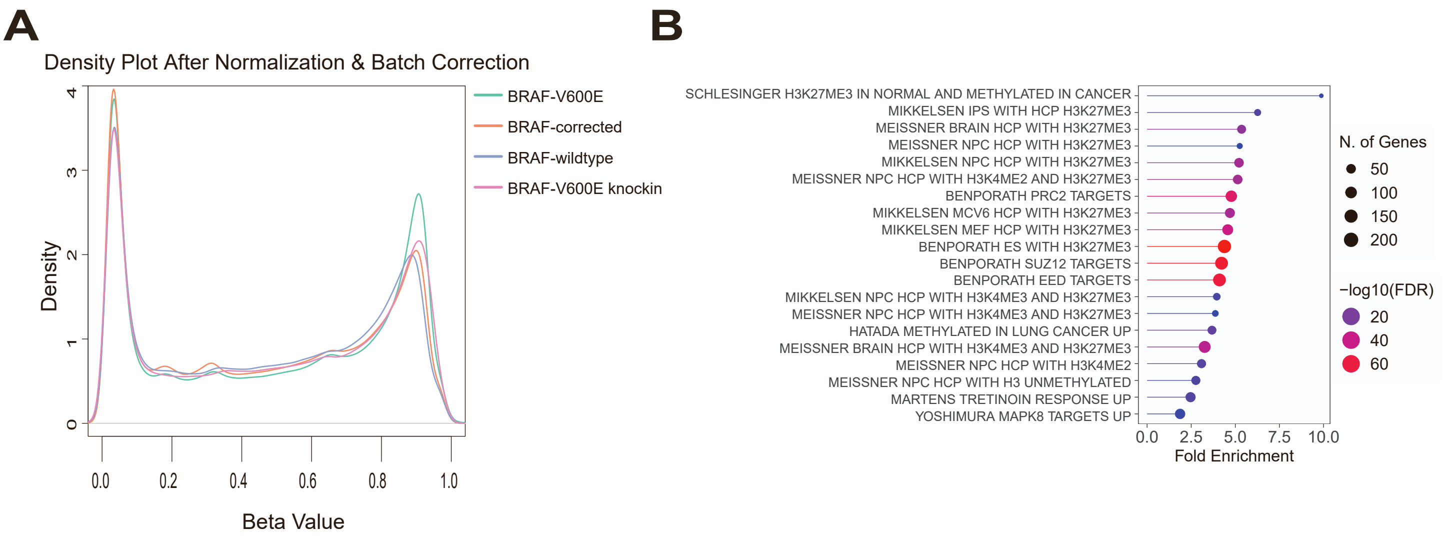

Figure S7: PRC2 target genes hypermethylation in HUB040-N-B organoids. (A) Density plot showing the distribution of B-values (methylation value: 0 = not methylated, 1 = fully methylated) measured at each DNA methylation probe in BRAF-V600E mutant (HUB040 and NC) and BRAF-corrected (BC1 and BC2), BRAF-wildtype (HUB040-N) and BRAF-V600E knock-in (HUB040-N-B) organoids. Two technical replicates per PDO were generated. (B) Gene-set analysis of hypermethylated CpG-annotated genes in HUB040-N-B, using gene sets curated in MSigDB (shinyGO platform), sorted on fold enrichment.

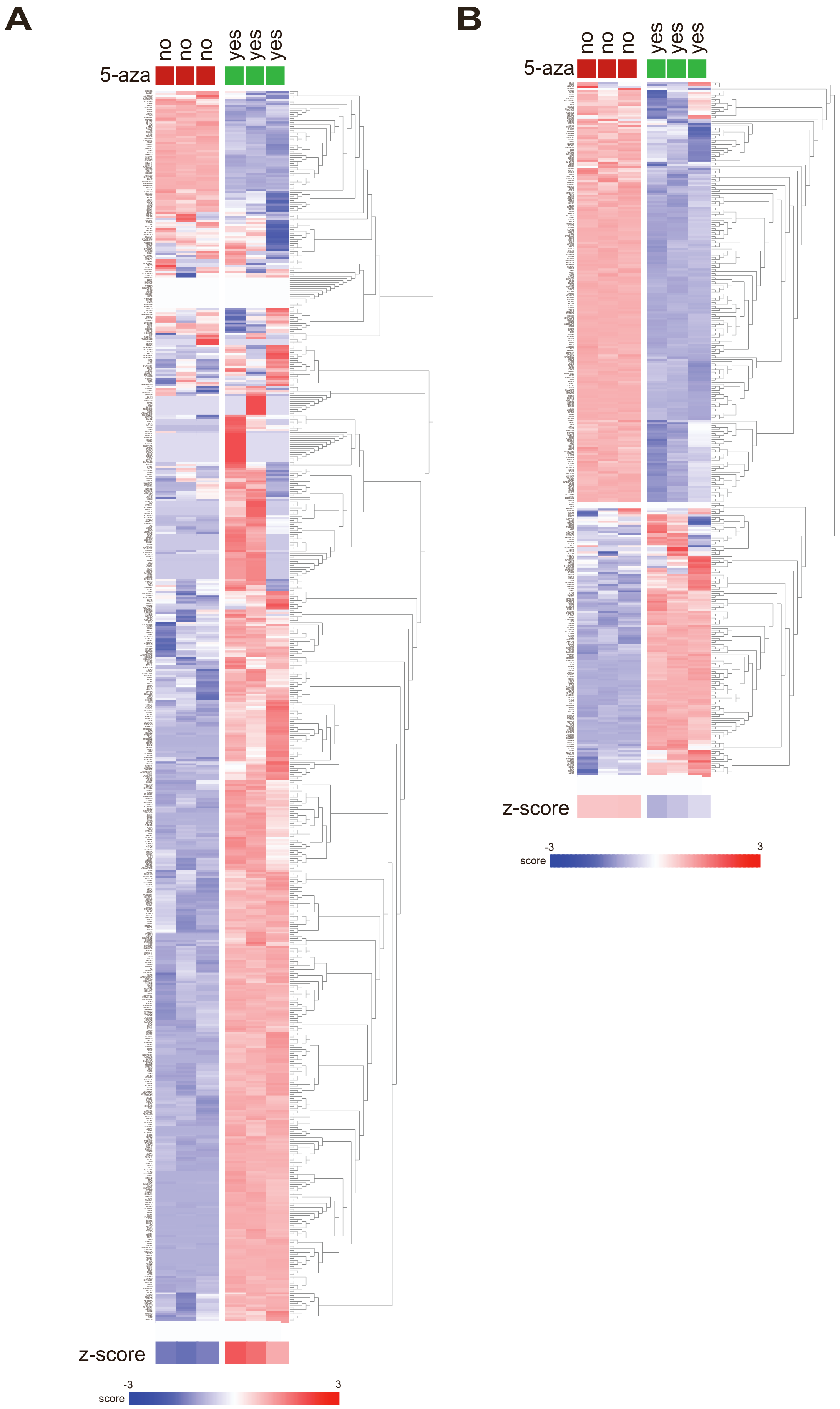

Figure S8: Expression of PRC2 targets and the ES signature in 5-aza versus non-treated BRAF-V600E mutant organoids. (A) PRC2 targets, (B) ES Signature in 5-aza treated HUB040 organoids.

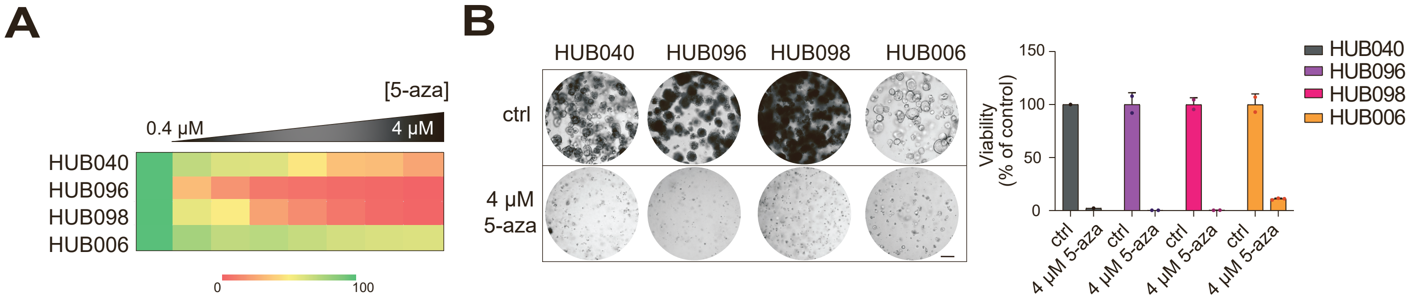

Figure S9: 5-aza treatment of BRAF-V600E mutant organoids results in durable treatment responses. (A) Heat map of dose-response measurements (logarithmic intervals) in BRAF-V600E mutant CRC PDOs (HUB040, HUB096, HUB098, and HUB006) to 5-aza-2'-deoxycytidine (5-aza) (10 days treated). Cell viability was measured using CellTiter-Glo 3D assays. Average of 3-6 technical replicates. (B) After 5-aza treatment, organoids were grown for seven additional days in CRC organoid growth medium to assess re-growth capacity. Pictures were taken at the end of the experiment. Cell viability was measured using CellTiter-Glo 3D assays. Data are presented as mean ± SD. Scale bar = 200 µM.

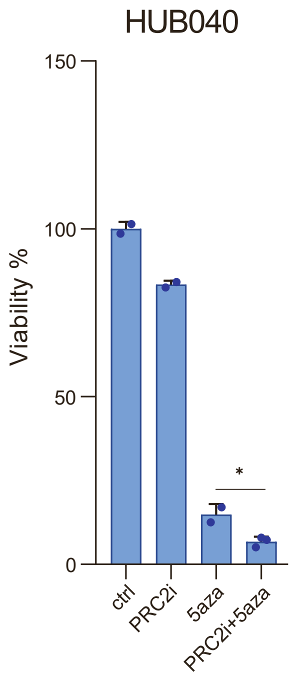

Figure S10: Combined inhibition of DNA methylation and PRC2-mediated H3K27me3 depositon.
Cell viability assay of BRAF-V600E mutant CRC PDO HUB040 treated for 10 days with 5-aza-2'-deoxycytidine (5-aza) (4 µM), PRC2 inhibitors (EED226 (4 µM) and GSK126 (4 µM)) or a combination of these treatments. Cell viability was measured using CellTiter-Glo 3D assays after 10 days. Average of 2-3 technical replicates. Data are presented as mean ± SD. *$P < 0.05$, one-way ANOVA with Tukey’s multiple comparison.
